# Microgravity and Hypergravity Induced by Parabolic Flight Differently Affect Lumbar Spinal Stiffness

**DOI:** 10.3389/fphys.2020.562557

**Published:** 2020-09-02

**Authors:** Jaap Swanenburg, Anke Langenfeld, Christopher A. Easthope, Michael L. Meier, Oliver Ullrich, Petra Schweinhardt

**Affiliations:** ^1^Integrative Spinal Research ISR, Department of Chiropractic Medicine, Balgrist University Hospital, Zurich, Switzerland; ^2^Cereneo Center for Interdisciplinary Research, Vitznau, Switzerland; ^3^Faculty of Medicine, Institute of Anatomy, University of Zurich, Zurich, Switzerland; ^4^Department of Machine Design, Engineering Design and Product Development, Institute of Mechanical Engineering, Otto-von-Guericke-University Magdeburg, Magdeburg, Germany; ^5^Space Medicine, Department of Industrial Engineering, Ernst-Abbe-Hochschule Jena, Jena, Germany; ^6^Zurich Center for Integrative Human Physiology (ZIHP), University of Zurich, Zurich, Switzerland

**Keywords:** stiffness, spine, microgravity, hypergravity, lumbar, parabolic flight

## Abstract

The objective of this study was to determine the response of the lumbar spinal motor control in different gravitational conditions. This was accomplished by measuring indicators of lumbar motor control, specifically lumbar spinal stiffness, activity of lumbar extensor and flexor muscles and lumbar curvature, in hypergravity and microgravity during parabolic flights. Three female and five male subjects participated in this study. The mean age was 35.5 years (standard deviation: 8.5 years). Spinal stiffness of the L3 vertebra was measured using impulse response; activity of the erector spinae, multifidi, transversus abdominis, and psoas muscles was recorded using surface electromyography; and lumbar curvature was measured using distance sensors mounted on the back-plate of a full-body harness. An effect of gravity condition on spinal stiffness, activity of all muscles assessed and lumbar curvature (*p*’s < 0.007) was observed (Friedman tests). *Post hoc* analysis showed a significant reduction in stiffness during hypergravity (*p* < 0.001) and an increase in stiffness during microgravity (*p* < 0.001). Activity in all muscles significantly increased during hypergravity (*p*’s < 0.001). During microgravity, the multifidi (*p* < 0.002) and transversus abdominis (*p* < 0.001) increased significantly in muscle activity while no significant difference was found for the psoas (*p* = 0.850) and erector spinae muscles (*p* = 0.813). Lumbar curvature flattened in hypergravity as well as microgravity, albeit in different ways: during hypergravity, the distance to the skin decreased for the upper (*p* = 0.016) and the lower sensor (*p* = 0.036). During microgravity, the upper sensor showed a significant increase (*p* = 0.016), and the lower showed a decrease (*p* = 0.005) in distance. This study emphasizes the role of spinal motor control adaptations in changing gravity conditions. Both hypergravity and microgravity lead to changes in spinal motor control. The decrease in spinal stiffness during hypergravity is interpreted as a shift of the axial load from the spine to the pelvis and thoracic cage. In microgravity, activity of the multifidi and of the psoas muscles seems to ensure the integrity of the spine. Swiss (BASEC-NR: 2018-00051)/French “EST-III” (Nr-ID-RCB: 2018-A011294-51/Nr-CPP: 18.06.09).

## Introduction

Astronauts who are exposed to microgravity often report lower back pain (LBP) that can significantly affect their ability to work (Sayson and Hargens, 2008). Total unloading of the spine, as happening in microgravity, also leads to deconditioning of the paraspinal muscles, flattening of the spinal curvature, and a change movement kinematics (Roll et al., 1998; Andreoni et al., 2000; Sayson and Hargens, 2008; Crevecoeur et al., 2010; Gaveau et al., 2011; Chang et al., 2016). It has been speculated that swelling of the intervertebral disks due to unloading is the underlying cause of LBP in astronauts (Sayson and Hargens, 2008). However, recent reports cast doubt on this, suggesting that rather than disk swelling, aberrant patterns in spinal stabilization mechanisms may be the main reason for pain (Chang et al., 2016; Bailey et al., 2018). Functional spinal stabilization is essential for spinal health, and is guaranteed by spinal motor control (Panjabi, 1992a; Cholewicki et al., 2000). Motor control of spinal musculature is a central aspect of these spinal stabilization mechanisms that have escaped the microgravity research focus to date. Spinal motor control is a complex system that combines different subsystems like the active, passive, and neural subsystems (Panjabi, 1992a). The passive subsystem mainly assures the end-range motion stability of the spine through the biomechanics of vertebra, facet joints, and interverbal disks (Arjmand and Shirazi-Adl, 2005). The active subsystem is based on the muscle system. With altered muscle tension and activity, the muscle system reacts by changing the force vector in relation to the spine (Bergmark, 1989). The neural subsystem combines the information from the passive (end of motion) and active subsystems (position and motion) to determine the status of the spine stability and reacts correspondingly to stabilize the spine (Frank et al., 2013). The human spine must withstand numerous external loads during the activities of daily life, even when simply walking or carrying objects (Liu et al., 2016). By contrast, the spine experiences relief from axial loading while sitting (Rohlmann et al., 2012) because the weight of the upper torso during sitting is partially held by the chair. Previous studies have investigated the dynamic behavior of the lumbar spine during changes in body position (Liu et al., 2016; Naserkhaki and El-Rich, 2017). Changing from a prone to an upright position increases spinal muscle activity to stabilize the spine to match the change in loading evoked through gravity (Chan et al., 2012; Swanenburg et al., 2018). In the prone position, the largest contributor to spinal stability is the inherent tension of passive muscle stiffness, ligaments, and joint capsules (Bergmark, 1989). In an upright position, including walking, standing, or sitting, a greater active control of spinal alignment, achieved through muscular activity, is needed (Bergmark, 1989). Total unloading of the spine, as experienced during space flight, presents a unique challenge to the spinal motor control system. The spinal system consists of different anatomical structures each with their own mechanical properties. Spinal stiffness as the net resistance on a macroscopic level to an externally induced deformation of the spinal system as a whole (Girod et al., 2003). In an earlier pilot study, we observed increased spinal stiffness during exposure to microgravity μg (MG) and decreased spinal stiffness during hypergravity 1.8 g (HG) exposure in parabolic flight maneuvers in a single test subject (Swanenburg et al., 2018). This was a surprising and counterintuitive result; we had expected that the spinal stiffness would decrease during MG due to the reduced load and increase during HG due to nearly double load. A possible explanation was that stiffness increase could be a reaction to the sudden change in gravity, which could have led to a safety co-contraction of the lumbar muscles that secured spinal integrity (Swanenburg et al., 2018). We subsequently confirmed this HG response pattern in a larger study with 100 young, healthy volunteers by investigating changes in spinal stiffness during axial loading of the lumbar and thoracic spine (Hausler et al., 2020). The results of these studies demonstrate the adaptability and complexity of the spinal motor control strategy and its dependency on differences in gravity or axial loading conditions. Whether the MG changes in spinal stiffness observed in a single subject are reproducible, and how they relate mechanistically to HG changes, remains unknown. Answering this might give insight into the pathophysiology of LBP in astronauts.

Therefore, the objective of the current study was to determine the response of the spinal motor control to MG and HG conditions. This was accomplished by testing the hypotheses that lumbar spinal stiffness, lumbar extensor and flexor muscles, and lumbar curvature change with different gravity conditions during parabolic flight. Spinal stiffness is the result of the integration of passive, active and neural motor control subsystems and thereby serves as a proxy measure of spinal motor control (Panjabi, 1992a). The curvature of the lumbar spine and muscle activity were measured as contributors to spinal stiffness and, in the case of muscle activity, as an index of the active sub-system (Edmondston et al., 1998; Rodriguez-Soto et al., 2013).

## Materials and Methods

### Participants and Parabolic Flight

Eight healthy participants (three females, five males, age 35 ± 9 years) participated in this study. Participants passed the required aviation medical screening during which neural or musculoskeletal disorders were excluded (Ullrich and Buhler, 2019) and provided written informed consent to participate in this study. Measurements were conducted at two different parabolic flight campaigns (PFC): the first two participants during the third Swiss PFC (VP 138) and six participants during the seventy-first European Space Agency (ESA) PFC mission (VP 143). Both PFCs were operated by Novespace, Bordeaux, France on board the Airbus A310 ZERO-G. The ethics committee of the Canton of Zurich approved third Swiss PFC study (BASEC-NR: 2018-00051). The French “Comite de protection des personnes EST-III” approved the seventy-first ESA PFC study (Nr-ID-RCB: 2018-A011294-51/Nr-CPP: 18.06.09).

### Experimental Design

A single-group repeated-measures design was used to measure changes in spinal motor control evoked by HG and MG. HG and MG were induced using parabolic flight. Parabolic flights offer a sequence of consecutive gravity conditions including 1 g Earth gravity (EG), 1.8 g HG, and μg MG, with small variations of the exact gravitational forces during the different gravity conditions. The course of one parabola started with a horizontal and level flight with normal Earth gravity (EG), followed by a steep climb flight that induced 20 s of HG (1.8 × g). Next, the airplane pushed over the top to begin 22 s of MG within the parabola. Subsequently, a second HG phase followed to return to normal flight level. Measurements were executed in upright posture. Before flight (30 min), participants were administered scopolamine (0.25 mg/1 mL; 0.7 mL for males and 0.5 mL for females) to prevent motion sickness (Spinks and Wasiak, 2011; Ritzmann et al., 2016). The administration of scopolamine does not interfere with sensorimotor skills associated with neuromuscular control (Ritzmann et al., 2016). One participant and the measurement operator had previously experienced MG and HG in parabolic flight. Blinding of the measurement operator was not possible.

### Measurement Setup

#### Stiffness

**FIGURE 1:**
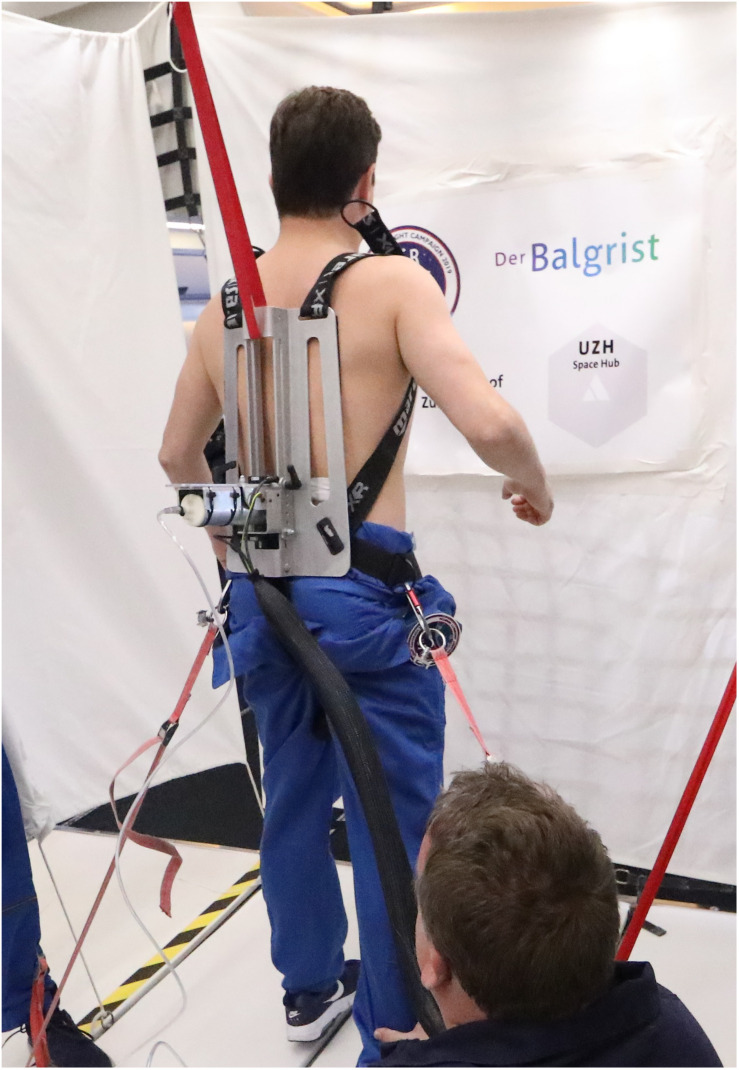
Measurements in microgravity during the 71st ESA Parabolic Flight Campaign 2019. Pic by Novespace.

**FIGURE 2:**
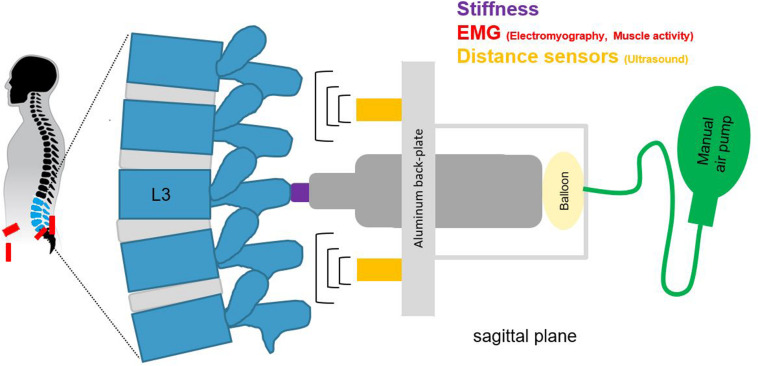
A schematic of the measurement set-up, for spinal stiffness, muscle activity, and lumbar curvature with two distance sensors.

Spinal stiffness for the purpose of this study was defined as the resistance to deformation of the spinal system, which includes passive rigid structures (bones), passive deformable structures (ligaments, disks), and active structures (muscles) (Panjabi, 1992a, b; Hodges et al., 2013; Needle et al., 2014). To measure this, we used an impulse head impactor mounted on an aluminum structure to which participants were strapped with a full-body harness. The “PulStar” a computer-a ssisted analytical device (Function Recording and Analysis System device PulStarFRAS, Sense Technology Inc., Pittsburgh, PA, United States) was mounted on the aluminum structure to measure the posterior-to-anterior spinal stiffness (Figure 1; Leach et al., 2003; Hofstetter et al., 2018; Hausler et al., 2020). This device generates an 80 N impulse, which is applied to the L3 spinous process (Swanenburg et al., 2018). A force transducer within the device transmits the impulse response to the measurement laptop. The impulse response quantifies the reaction of the muscles, joints and ligaments to the energy infused by the impulse (Leach et al., 2003) and is thus a proxy for spinal stiffness. The reaction of the involved tissues can be approximated with a linear, time-invariant system that is disturbed by a very brief (< 1 ms) input signal (impulse). Within this framework, the impulse response completely characterizes the reaction (Girod et al., 2003) and can be reported as a force (Newton) with no change in time. A manual air-pump was used to compress the impulse head preload spring until the preload criteria of 18 N was met and the instrument generated the measurement impulse. Preloading was applied to minimize the influence of soft tissue components between the device and spinous process (Swanenburg et al., 2018). After the measurement, the valve on the air pump was opened so that the air could escape and the device returned to its starting position. To ensure that the impulse head was aligned precisely with the spinous process of L3, a portable ultrasound device (Aloka SSD-500 and Aloka UST-934N-3.5 Electronic Convex Probe; Aloka Co., Tokyo, Japan) was used (Hausler et al., 2020). Figure 2 shows schematic of the measurement set-up.

#### Muscle Activity

One way to describe the muscular balance of the human spine is to differentiate between local and global muscle systems. The local system consists of muscles with insertion and/or origin at the lumbar vertebrae, the global system of muscles with origin at the pelvis and insertion at the thorax (Bergmark, 1989). Here, muscles of both systems were assessed. Specifically, the erector spinae and multifidi muscles were assessed for the local system and the transversus abdominis muscle for the global system. Additionally, the activity of the psoas muscle was measured because a of possible contribution to lumbar stabilization (Juker et al., 1998). Muscle activity of the erector spinae, multifidi, transversus abdominis, and psoas muscles (McGill et al., 1996; Jiroumaru et al., 2014) was recorded using surface electromyography (EMG). Of note, crosstalk by surrounding muscles groups might contribute to the muscle activity recorded for the multifidi (Stokes et al., 2003). However, because the focus of the present study is the assessment of possible changes in motor control of back muscles with similar function, this is not considered a major issue. For simplicity, ‘multifidus activity‘ is used in the following. For each muscle on both sides, the skin was preconditioned using a combination of skin razor, abrasive gel (NuPrep; Weaver and Company, Aurora, CO, United States) and alcohol to remove skin residue. Subsequently, two Ag/AgCl gel electrodes (H124SG; Covidien, Wapole, MA, United States) were applied with an intra-electrode distance of 35 mm on the muscle belly according to the Surface ElectroMyoGraphy for the Non-Invasive Assessment of Muscles (SENIAM) guidelines. One exception was the psoas muscle, for which the electrode locations were determined using ultrasonography to locate the muscle within the inguinal region between the sartorius muscle and the femoral neurovascular bundle (Katsavrias et al., 2005). Wireless surface EMG transmitters (pico/aktos; Myon AG, Schwarzenberg, Switzerland) with integrated accelerometers were attached to the electrode pairs. The accelerometers served to measure the exact gravitational forces throughout the experiment. The EMG (2000 Hz) and accelerometer signals (148 Hz) were prefiltered (EMG, Bandpass 10–500 Hz; Accelerometer, Bandpass 1–70 Hz) and recorded by a measurement laptop. The locations of the electrodes were marked before the flight on the ground by two team members to assure correct positioning. Afterward the electrodes were put into place and, to avoid displacement, secured by tape. The signal quality was assessed before the flight by checking the data for noise and artifacts right after they were put in place. This procedure was repeated before data collection started during the flight to check for correct functioning of the electrodes. Placement of the electrodes can be found in Supplementary Figures S2, S3.

#### Lumbar Curvature

Lumbar curvature was assessed by two sensors mounted on the aluminum back-plate of the full-body harness. The ultrasonic distance sensors (UC250-F77-IU-IO-V31; Pepperl + Fuchs, Mannheim, Germany) were integrated into the backplate at + 2 cm rostrally (upper sensor) and −2 cm caudally (lower sensor) to the stiffness device (Figures 1, 2). These sensors measured the perpendicular distance between the back-plate and the skin. Given that the back-plate is rigid, any increase in distance measured by the sensors reflects an increase in lumbar curvature. The sensors were synchronized with the impactor before each measurement and recorded distance data onto the measurement laptop continuously throughout the flight at 140 Hz.

#### Measurements in Changing Gravity

Per participant, a sequence of 15 parabolas was performed. The first and second parabolas were conducted to familiarize the participants with different gravity conditions and to minimize anxiety. During these first parabolas, the measurement operator secured the participants; therefore, no stiffness measurements were conducted. The final parabola was likewise discarded, as participants were fatigued and already preparing to exit the measurement apparatus. This resulted in a dataset consisting of 12 parabolas per participant. Because respiration can affect spinal stiffness measurements, the participants were instructed to hold their breath at the end of a normal exhalation for the measurement (Shirley et al., 2003). Tethers between the harness and the aircraft prevented participant drifting away in MG (Swanenburg et al., 2018).

### Data Preparation

Muscle activity data was processed in MATLAB (2019b, Mathworks, Natick, MA, United States). Using the mean left and right vertical accelerometer traces of the erector spinae, gravitational steady states (EG, 0.9–1.1 g; HG, 1.7–1.9 g; MG, −0.1–0.1 g) were identified for each parabola (n_parabola_ = 12). Thereafter, the shortest duration of all detected steady states in all participants was determined, which was used as the data extraction window (t_SteadyState_ = 11.4 s). All EMG channels were segmented into the gravitational steady states, and signals of t_SteadyState_ duration were extracted for each participant, parabola, and muscle. Extraction algorithmically attempted to maximize the distance of this window from any state change while minimizing spectral and raw signal variance. Each sequence was visually verified before calculating the root mean square (RMS). In the case of visible aberrant spectral content or raw signal artifacts in the raw signal, windows were manually shifted to reduce these. For each parabola, RMS of the first EG, MG, and HG state were normalized to the average RMS of EG1 and EG2 states. The resultant ratio was used for further analysis. The normalization to average EG was performed within each parabola individually, as there was large inter-parabola variance in the RMS extracted for the EG states. Within-parabola, the two EG states demonstrated good agreement. Finally, for each muscle and participant, the relative RMS ratio was averaged over all twelve parabolas and for left and right sides. This resulted in a single average RMS value for each muscle and participant. This method was selected in order to avoid averaging raw EMG signals from all parabolas – an approach that could result in contamination of the RMS through microvariations of the gravitational steady state (± 0.1 g). An example of raw data is found in Supplementary Figure S4. Values from the ultrasonic distance sensors and from the spinal impactor were used as provided by the sensors without preprocessing.

### Data Analysis

We used the Shapiro–Wilk test to test data for normal distribution. In case of a non-normal data distribution differences across all three gravity conditions were analyzed using the Friedman Test. For *post hoc* comparisons relative to 1 g, a Wilcoxon signed-rank test was used. The significance level was set at *p* < 0.05. For EMG measurements of the four muscles, Bonferroni correction for multiple comparison was used and thus, *p* < 0.0125 were considered significant. Effect sizes were calculated with the formula ES = Z/√N where Z corresponds to the Wilcoxon-test output and N to the number of observations. Effects sizes are interpreted as follows: small effect < 0.3; moderate < 0.5; ≥ 0.5 large effects (Coolican, 2009). Power was calculated *post hoc* with G^∗^power (Faul et al., 2007) for the *post hoc* tests (Wilcoxon) of the *t*-tests family (α = 0.05, sample size *N* = 8, effect size). The data was transferred, stored, and analyzed using the IBM SPSS 25 statistical software package (SPSS Inc., Chicago, IL, United States).

## Results

### Spinal Stiffness

**FIGURE 3:**
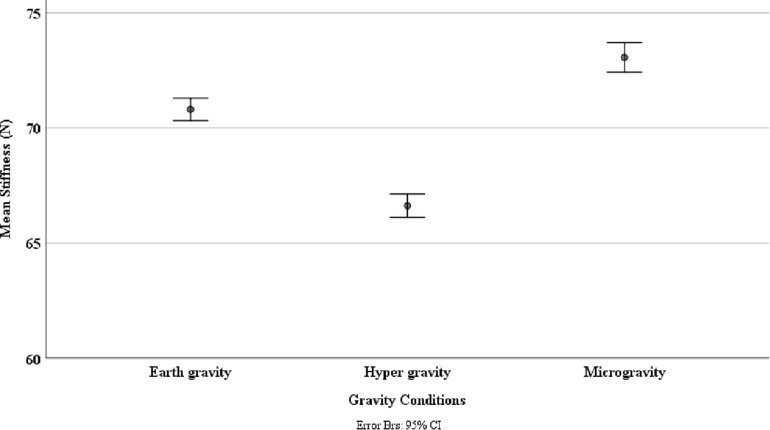
Spinal stiffness between different gravity conditions: earth gravity, hypergravity, and microgravity. This was measured via an impulse head impactor mounted on an aluminum structure to which participants were strapped with a full-body harness.

Shapiro–Wilk testing indicated that the data were not normally distributed. The Friedman test showed a statistically significant influence of gravity conditions on spinal stiffness [χ^2^(2) = 196.395; *p* < 0.001]. Results are shown in Figure 3. *Post hoc* analysis showed significant reduction in stiffness during HG (*p* < 0.001) and increase in stiffness during MG (*p* < 0.001).

### Muscle Activity

There was a significant effect of gravity condition on all muscles assessed: erector spinae [χ^2^(2) = 31.122; *p* < 0.001]; multifidi [χ^2^(2) = 52.231; *p* < 0.001], transversus abdominis [χ^2^(2) = 151.185; *p* < 0.001], and psoas [χ^2^(2) = 63.352; *p* < 0.001]. *Post hoc* analysis showed significant increase in muscle activity in all muscles during HG (*p* < 0.001). During MG the multifidi (*p* < 0.002) and transversus abdominis muscle activity (*p* < 0.001) increased significantly. In contrast, no significant change of the psoas (*p* = 0.850, *r* = −0.413) and erector spinae (*p* = 0.813) muscle activity was found during MG.

### Lumbar Curvature

**FIGURE 4:**
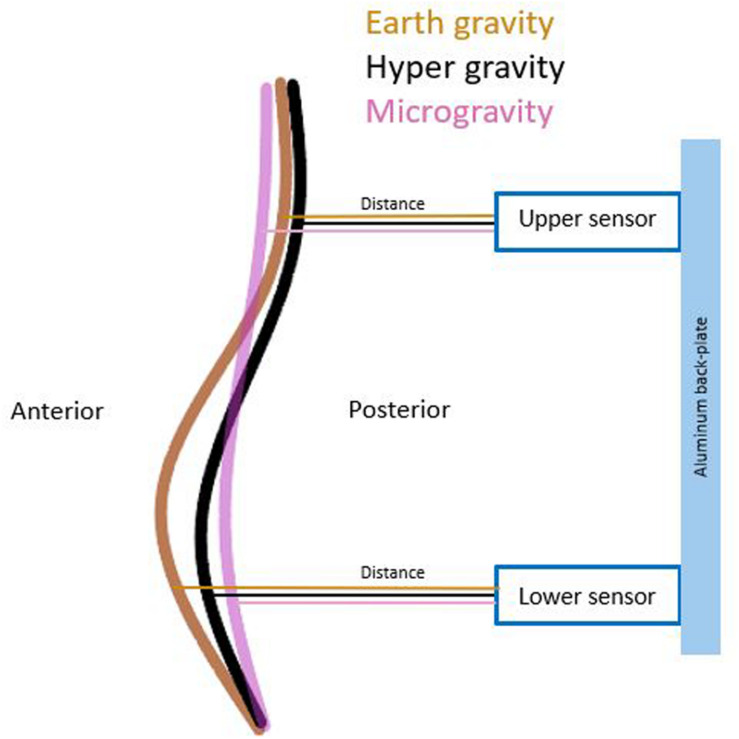
A schematic of the change in distances of both sensors across the different g conditions.

Gravity had significantly effect on both lumbar distances [upper sensor: χ^2^(2) = 9.957, *p* = 0.007 lower sensor: χ^2^(2) = 10.344, *p* = 0.006]. *Post hoc* analysis showed a significant decrease in distance in the upper (*p* = 0.016) and lower (*p* = 0.036) sensors during HG. During MG, the upper sensor showed a significant increase (*p* = 0.016), and the lower showed a decrease (*p* = 0.005) in distance. A schematic of the changed distances across conditions between sensors and lumbar back are shown in Figure 4. All mean values and *post hoc* analysis, effect sizes, and power calculations are shown in Table 1.

**TABLE 1:** Mean values, *post hoc, effect size, and power* results of spinal stiffness, muscle activity, and lumbar curvature during earth gravity, hypergravity, and microgravity.

	EG Mean ± SD	HG Mean ± SD	MG Mean ± SD	EG–HG				EG–MG			
				Z	*p*	ES	P	Z	P	ES	P
**Spinal Stiffness (*N*)**											
	70.81 ± 5.20	66.62 ± 5.18	73.7 ± 6.76	–10.497	< 0.001*	–2.624	0.999	–4.503	< 0.001*	–1.126	0.871
**Muscle activity**	**(#)**	**(#)**	**(#)**								
Erector spinae	0.93 ± 0.20	1.31 ± 0.47	0.98 ± 0.50	–6.611	< 0.001*	–1.652	0.992	–1.190	0.850	–0.413	0.268
Multifidi	0.95 ± 0.17	1.49 ± 0.49	1.14 ± 0.55	–7.913	< 0.001*	–1.978	0.999	–3.029	0.002^#^	–0.757	0.594
Transversus abdominis	0.92 ± 0.14	1.35 ± 0.32	0.62 ± 0.34	–8.938	< 0.001*	–2.235	0.999	–6.737	< 0.001^#^	–1.684	0.993
Iliopsoas	0.98 ± 0.18	1.28 ± 0.61	1.03 ± 0.39	–6.851	< 0.001*	–1.713	0.995	–0.236	0.813	–0.059	0.067
**Lumbar curvature (mm)**											
Upper distance	61.18 ± 11.91	60.12 ± 11.98	62.58 ± 12.95	–2.408	0.016*	–0.602	0.440	–2.408	0.016*	–0.602	0.440
Lower distance	73.37 ± 12.61	72.87 ± 13.28	71.39 ± 12.21	–2.094	0.036*	0.523	0.364	–2.799	0.005*	–0.700	0.536

EG, earth gravity; HG, hypergravity; MG, microgravity; SD, standard deviation; N, newton; mm, millimeter; Z/p, Wilcoxon signed rank test. *p < 0.05 (significant); ^#^p < 0.0125 (significant with Bonferroni correction); ES, Effect size; #, RMS standardized to the average of the preceding and subsequent EG states; P, Power.

## Discussion

This study investigated the role of spinal motor control strategies maintaining lumbar spine integrity during changing gravity conditions. Spinal stiffness increased during HG and decreased during MG, confirming the differential changes in spinal stiffness during HG and MG previously observed in a single-case study (Swanenburg et al., 2018). Spinal stiffness measures were complemented in the present study by measuring lumbar curvature and muscle activity.

### Hypergravity

Concurrently with the decrease in lumbar spinal stiffness during HG, an increase in lumbar muscle activity and a flattening of the lumbar curvature occurred. Flattening of the lumbar curvature during HG corroborates findings of a study in which participants were carrying heavy backpacks of up to 60% of their body weights (Rodriguez-Soto et al., 2013). Both, flattening of the lumbar curvature as well as muscle activity of the local and global systems have differential effects on spinal stiffness. Lumbar curvature flattening by itself would increase spinal stiffness because lumbar stiffness increases when the lumbar spine is moved away from its neutral position (Edmondston et al., 1998). With regard to muscle activity, increased activity of the local system, i.e., the muscles attaching to the lumbar vertebrae, would likely lead to increased spinal stiffness because the local system provides local mechanical stability of the lumbar spine (Shirley et al., 1999; Stokes and Gardner-Morse, 2003). In contrast, activation of the global system leads to a load shift away from the spine toward the thoracic cage and the pelvis (Bergmark, 1989); thereby decreasing spinal stiffness (Swanenburg et al., 2018; Hausler et al., 2020). Because the *in vivo* assessment of spinal stiffness as performed here represents the net effect of all subsystems, it appears that the effects of the increased activation of the global muscle system dominated over the effects of the local muscle system and the flattening of the lumbar spine. Such dominance of the global system, which supports the global mechanical model of the spinal system by Bergmark (1989), would be achieved by a change in spinal motor control. Table 2 depicts a model integrating the net effect on spinal stiffness observed here with the effects of the different subsystems on spinal stiffness described in the literature.

**TABLE 2:** Model to explain observed spinal stiffness by the expected contributions of the subsystems during HG.

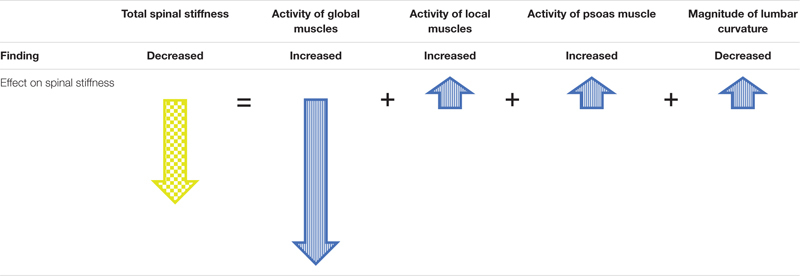

*Yellow, measured; Blue, expected; Finding, findings of the present study.*

### Microgravity

MG led to an increase in lumbar spinal stiffness, an increase in multifidi muscle activity, a decrease in transversus abdominis muscle activity, and a flattening of the lumbar curvature. In contrast to the HG condition, no change in muscle activity in the erector spinae and the psoas was detected.

As discussed in “Hypergravity.” the decrease of spinal stiffness during HG can be explained by the local and global mechanical model of the spinal system. Similarly, the increase in spinal stiffness during MG can be explained by the observed activity increase of a local stabilizer, the multifidus muscle, and the observed activity decrease of a global stabilizer, the transversus abdominis muscle. In contrast, no change of the activity of the psoas and the erector spinae muscles was detected during MG. Therefore, the reaction of the muscles measured during the present study fits only partially to the local and global mechanical model of the spinal system. During long exposure to MG, e.g., during space missions, the erector spinae shows a similar reduction in activity and muscle mass degeneration as other spinal muscles (LeBlanc et al., 2000; Chang et al., 2016). Thus, it is possible that the erector spinae differentiates itself by not quickly adapting in activity during short or sudden MG conditions, such as a parabolic flight. In the original local and global mechanical model by Bergmark (1989), the psoas muscle was not included in the local system, despite its segmental spinal attachments (Bergmark, 1989). Interestingly, activity of the psoas muscle neither changes nor does it degenerate during prolonged exposure to an MG environment, in contrast to all other muscles of the lumbar spine that have been studied (Chang et al., 2016). Therefore, the psoas muscle may act like a local stabilizer during short and long durations of MG. MG itself reduces lumbar curvature (Andreoni et al., 2000), as also observed here, and the psoas muscle might stabilize the lumbar spine by adapting its activity to the current degree of the lumbar curvature (Penning, 2000), meaning that the psoas muscle would have to constantly activated, plausibly leading to muscle fatigue and pain (Granata et al., 2004). Indeed, iliopsoas tightness is associated with pain in the lumbar area (Johnston et al., 1998; Barker et al., 2004), which is often relieved by sitting (Bachrach, 1988). Sitting is not possible during space flight but similar relaxation of the psoas muscle can be achieved by the fetal tuck position. Some astronauts report an improvement of LBP when taking up such a position (Penning, 2000). Based on these considerations, we have previously suggested that the psoas muscle might play a particularly important role in the development of LBP in astronauts (Swanenburg et al., 2018).

Similar to HG, a small flattening of the lumbar curvature occurred during MG, which might have contributed to the observed increase in spinal stiffness. However, it occurs that the shape of the flattening differs between HG and MG. During HG, both sensors showed a decrease in distance, which is expected by the load shift away from the spine by the dominance of the global stabilizers. In contrast during MG, the lower sensor showed a decrease whereas the upper showed an increase in distance. This is expected with the largely reduced gravitational weight that leads to an overall flattening of all spinal curvatures (Sayson and Hargens, 2008). A model of direction of change observed, which describe the result of total spinal stiffness in MG, are shown in Table 3. The influence of the passive structures such as the vertebra or facet joints on spinal stiffness during HG and MG conditions is assumed to be very low because they contribute very little to stability in the neutral zone (Arjmand and Shirazi-Adl, 2005). Because the observed change in the curvature of the lumbar spine was small, the passive structures of the spine stayed within the neutral zone throughout the measurements. Therefore, in both MG and HG, the influence of the passive structures on spinal stiffness is deemed neglectable.

**TABLE 3:** Model to explain observed spinal stiffness by the expected contributions of the subsystems during MG.

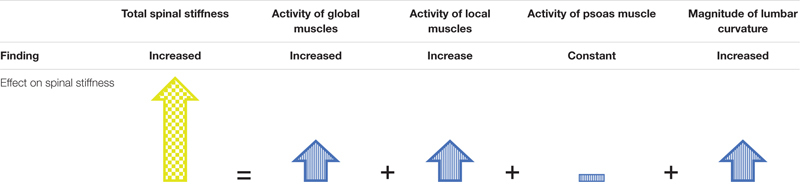

*Yellow, measured; Blue, expected; Finding, findings of the present study.*

### Limitations

In this study, the spinal curvature was measured with distance sensors at two locations of the lumbar spine. Because only two points were measured, these measurements provide a limited assessment of the spinal curvature. Although significant changes were observed, more measurement sites should be used in future studies to obtain more detailed information about curvature changes. Also, it might be argued that the psoas EMG signal is contaminated with crosstalk from the sartorius muscle, however, this has been previously quantified as negligible with appropriate electrode placement (Jiroumaru et al., 2014).

## Conclusion

This study emphasizes the role of adaptations of spinal motor control under changing gravity conditions. Both HG and MG lead to changes in spinal motor control. During HG, the axial load appears to shift from the spine to the pelvis and thoracic cage, whereas in MG, ongoing activity of the erector spinae muscle and of the psoas muscle seems to ensure the integrity of the spine. Furthermore, when astronauts return to earth, their motor control must be re-adapted back to EG. This suggests that exercises for spinal motor skills should also be performed under different axial load situations as they occur in everyday life to reduce possible risk of injury to the spine.

## Data Availability Statement

The raw data supporting the conclusions of this article will be made available by the authors, without undue reservation, to any qualified researcher.

## Ethics Statement

The studies involving human participants were reviewed and approved by the Ethics Committee of the Canton of Zurich, Switzerland (BASEC-NR: 2018-00051), and the French Comite de Protection des Personnes EST-III (Nr-ID-RCB: 2018-A011294-51/Nr-CPP: 18.06.09). The patients/participants provided their written informed consent to participate in this study.

## Author Contributions

JS and PS developed the research questions and the design. JS and AL conducted the data acquisition. JS and CE carried out the analysis and interpretation of the results. JS produced an early version of the manuscript. JS, AL, MM, CE, OU, and PS revised the manuscript to bring it to its current version. All authors contributed to the article and approved the submitted version.

## Conflict of Interest

The authors declare that the research was conducted in the absence of any commercial or financial relationships that could be construed as a potential conflict of interest.
