# Supplementary material for: Microgravity and Hypergravity Induced by Parabolic Flight Differently Affect Lumbar Spinal Stiffness
Source: Front Physiol. 2020 Sep 2;11:562557. doi: 10.3389/fphys.2020.562557 (PMC7492749; doi:10.3389/fphys.2020.562557)

Supplementary Material

Figure S1 The measurement setup with stiffness device (SD) and the two ultrasonic distance sensors

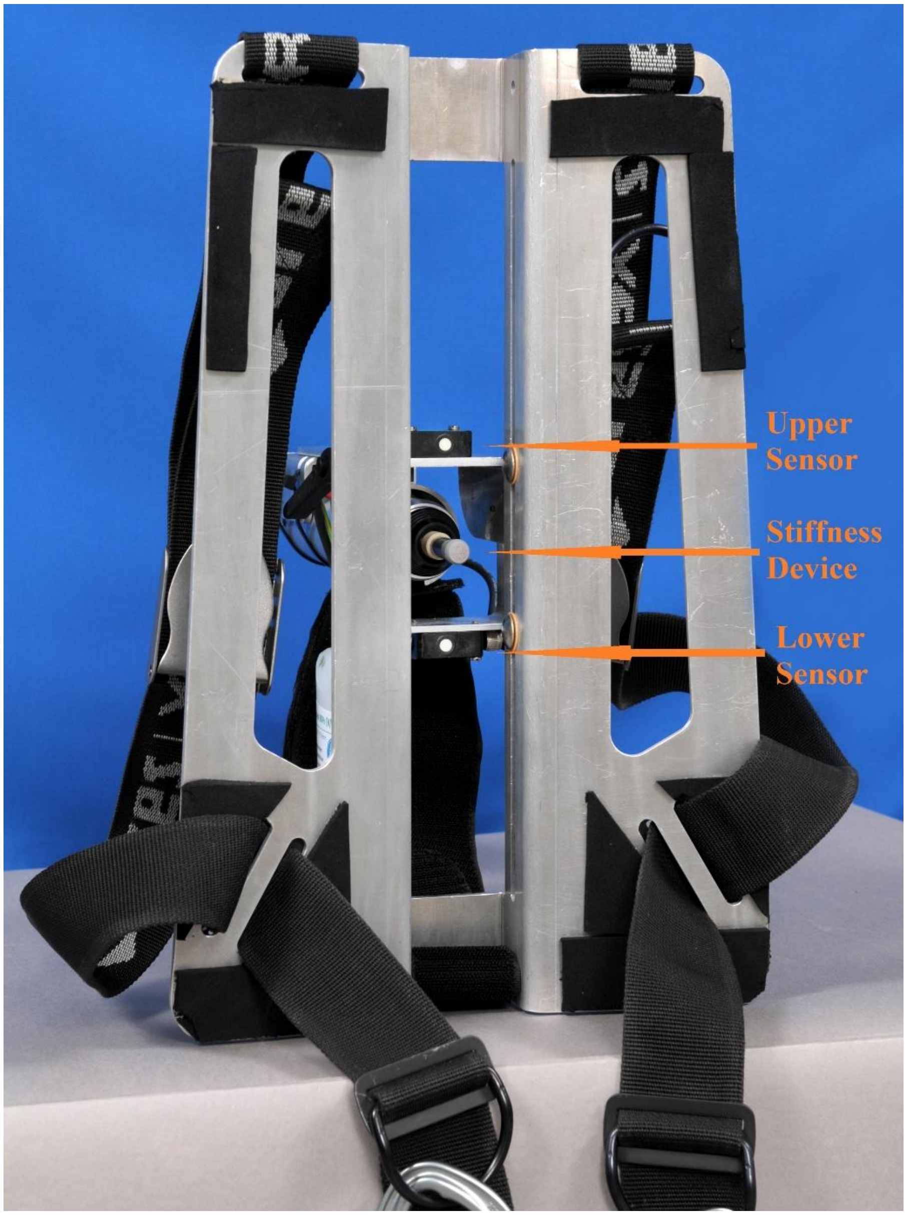

Figure S2 Placement of the electrode's at erector spinea and multifidus muscle

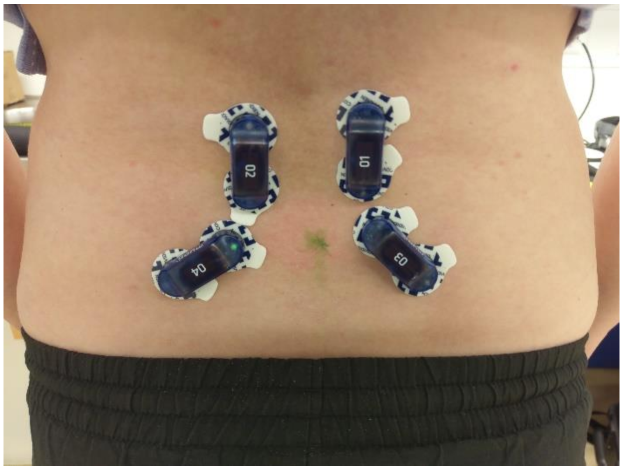

Figure S3 Placement of the electrodes at transversus abd. and iliopsoas muscle

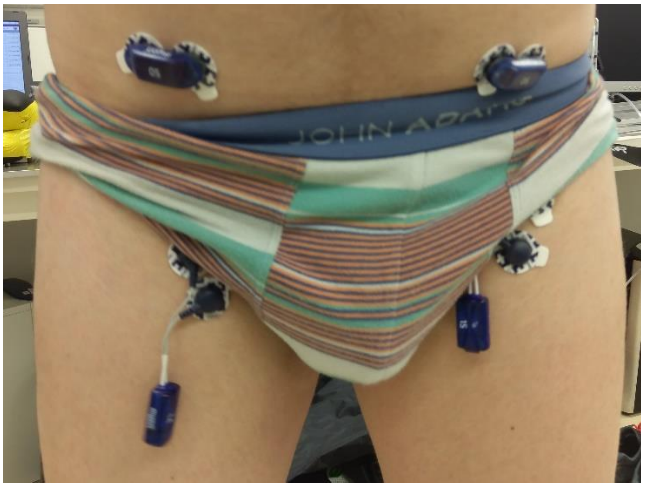

Figure S4 An exemplar of EMG raw data

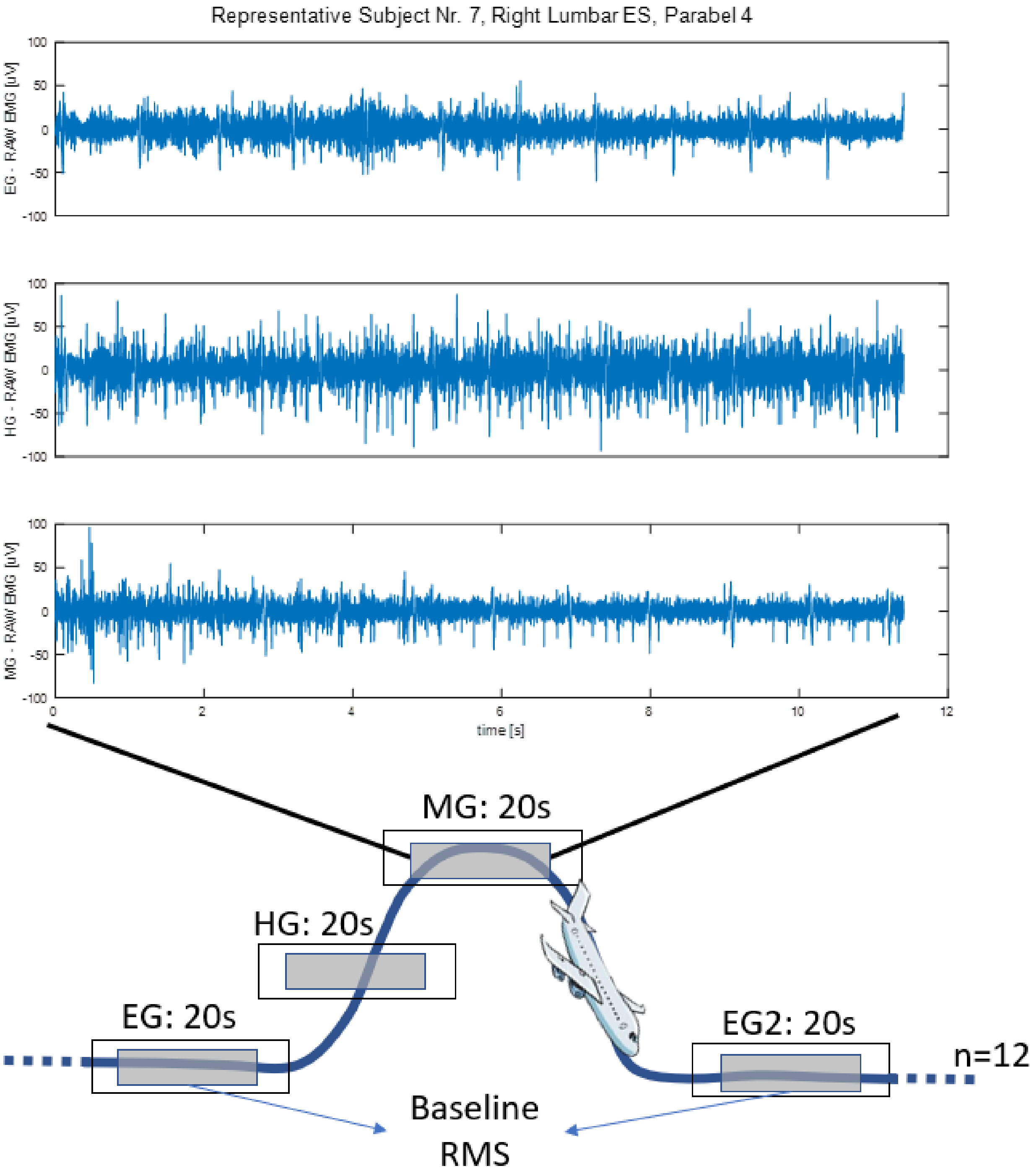
Representative Subject Nr. 7, Right Lumbar ES, Parabel 4
EG - RAW EMG [µV]
HG - RAW EMG [µV]
MG - RAW EMG [µV]
time [s]
MG: 20s
HG: 20s
EG: 20s
EG2: 20s
n=12
Baseline
RMS

Figure S5a Change of erector spinea muscle activity due to change in conditions

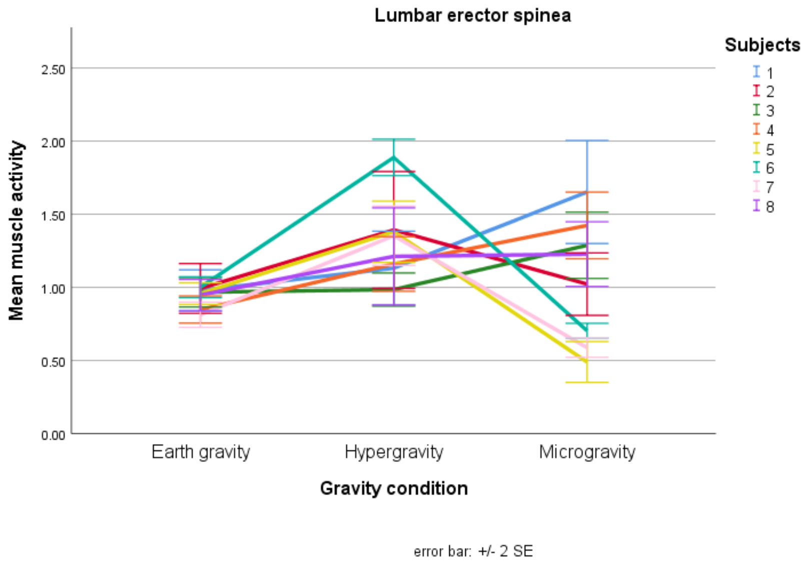

Figure S5b Change of multifidi muscle activity due to change in conditions

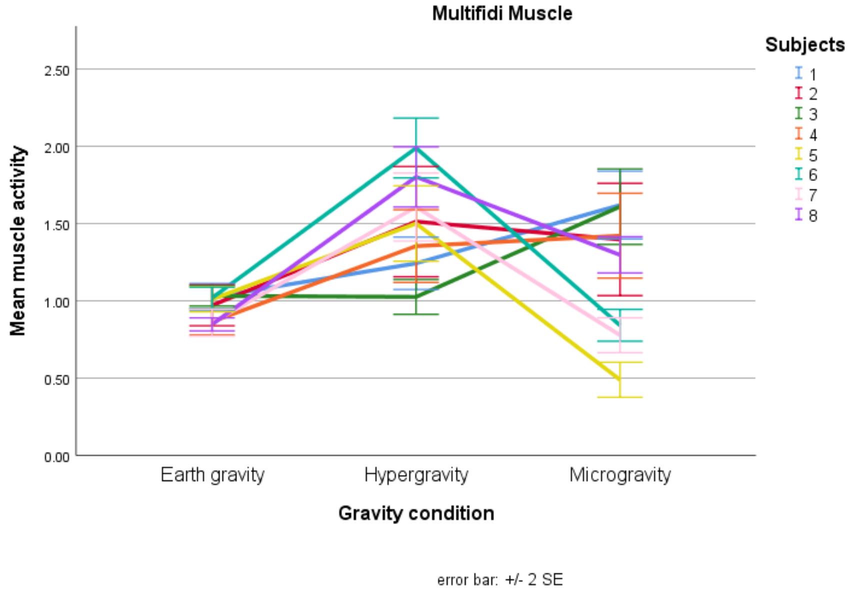

Figure S5c Change of transversus abdominis muscle activity due to change in conditions

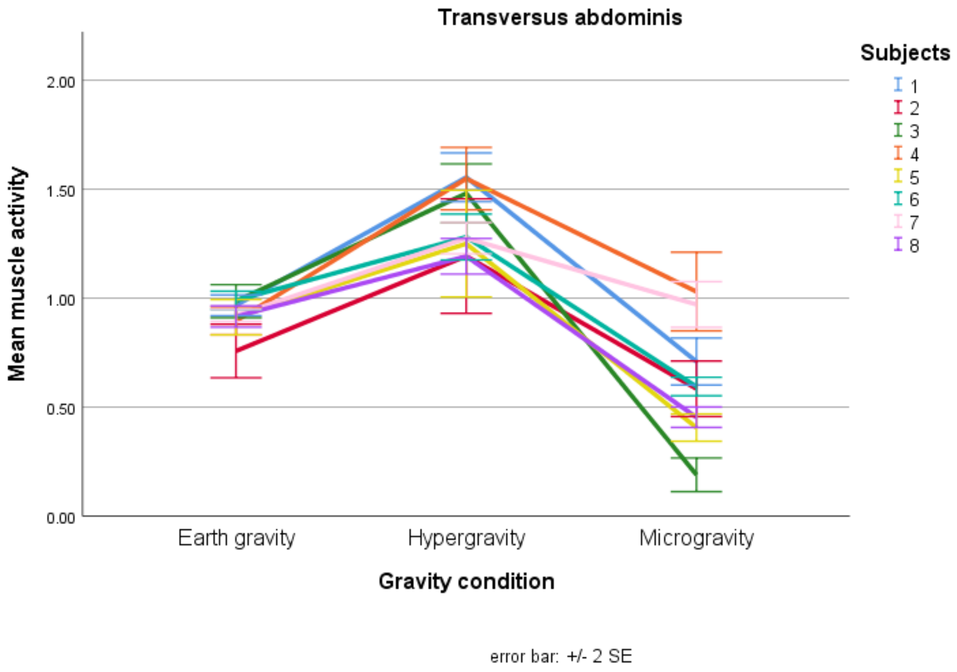

Figure S5d Change of psoas muscle activity due to change in conditions

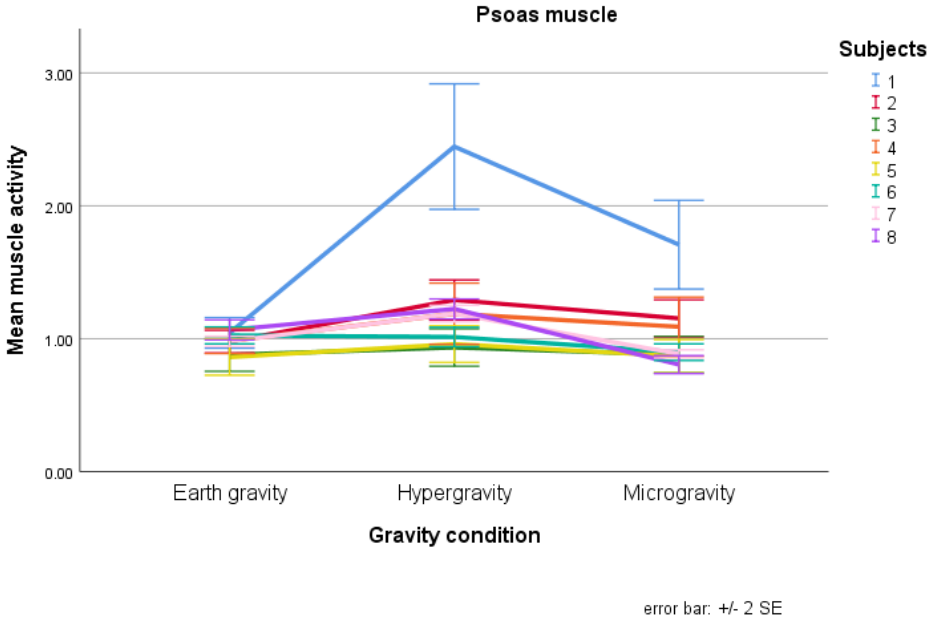

Supplement: Supplementary file 1 [file Data_Sheet_1.PDF]
